# Identification and characterization of porcine Rotavirus A in Chilean swine population

**DOI:** 10.3389/fvets.2023.1240346

**Published:** 2023-11-02

**Authors:** Victor Neira, Cristián Melgarejo, Constanza Urzúa-Encina, Felipe Berrios, Valentina Valdes, Sunil Mor, Barbara Brito-Rodriguez, Galia Andrea Ramirez-Toloza

**Affiliations:** ^1^Departamento de Medicina Preventiva Animal, Facultad de Ciencias Veterinarias y Pecuarias, Universidad de Chile, Santiago, Chile; ^2^Veterinary Population Medicine, College of Veterinary Medicine, University of Minnesota, Saint Paul, MN, United States; ^3^Department of Veterinary and Biomedical Sciences, Animal Disease Research and Diagnostic Laboratory, South Dakota State University, Brookings, SD, United States; ^4^Department of Primary Industries, Veterinary Research Office, Menangle, NSW, Australia

**Keywords:** Rotavirus A, pig, NGS, genotyping, zoonoses

## Abstract

Rotavirus A (RVA) is a common cause of diarrhea in newborn pigs, leading to significant economic losses. RVA is considered a major public health concern due to genetic evolution, high prevalence, and pathogenicity in humans and animals. The objective of this study was to identify and characterize RVA in swine farms in Chile. A total of 154 samples (86 oral fluids and 68 fecal samples) were collected, from 22 swine farms. 58 (38%) samples belonging to 14 farms were found positive for RVA by real-time RT-PCR. The samples with low Ct values (21) and the two isolates were selected for whole genome sequencing. Nearly complete genomes were assembled from both isolates and partial genomes were assembled from five clinical samples. BLAST analysis confirmed that these sequences are related to human and swine-origin RVA. The genomic constellation was G5/G3-P[7]-I5-R1-C1-M1-A8-N1-T1-E1-H1. Phylogenetic analysis showed that VP4, VP1, VP2, NSP2, NSP3, NSP4, and NSP5 sequences were grouped in monophyletic clusters, suggesting a single introduction. The phylogenies for VP7, VP6, VP3, and NSP1 indicated two different origins of the Chilean sequences. The phylogenetic trees showed that most of the Chilean RVA sequences are closely related to human and swine-origin RVA detected across the world. The results highlight the potential zoonotic nature of RVA circulating in Chilean swine farms. Therefore, it is important to continue RVA whole genome sequencing globally to fully understand its complex epidemiology and early detection and characterization of zoonotic strains.

## 1. Introduction

Rotavirus A (RVA), a member of the Sedoreoviridae family, genus Rotavirus, is a non-enveloped, icosahedral virus measuring approximately 80 nm in diameter (1). It possesses a triple capsid structure (1, 2). RVA is known to be a causative agent of severe intestinal disease and a leading cause of mortality of 200,000 people per year, especially in children under 5 years of age (3, 4). Furthermore, RVA is commonly associated with diarrhea in farm animals. In swine populations, it is a prevalent pathogen causing gastroenteritis, particularly in newborn pigs (5). The transmission of RVA occurs through the fecal-oral route, resulting in the shortening of villi, sparse and irregular microvilli, and mononuclear cell infiltration of the lamina propria of small intestinal enterocytes (6).

The genome of RVA consists of 11 linear segments of dsRNA that encode 12 structural and non-structural proteins (2). Due to its high prevalence and pathogenicity in both humans and animals, RVA is considered a significant public health concern (4, 7). Traditionally, the genotyping of RVA has been based on the structural proteins VP7 and VP4, denoted as Gx-P[x]. Currently, there are 42G genotypes and 58 P genotypes identified (8).^1^ However, for the comparison of rotavirus genomes, the classification Gx-P[x]Ix-Rx-Cx-Mx-Ax-Nx-Tx-Ex-Hx is commonly employed, representing the VP7-VP4-VP6-VP1-VP2-VP3-NSP1-NSP2-NSP3-NSP4-NSP5/6 genes, respectively (9, 10). This comprehensive genome comparison enhances our understanding of RVA viral dynamics and aids in comprehending transmission and spread.

Rotavirus A exhibits considerable genetic diversity in swine, with numerous G and P genotypes identified worldwide. Twelve G genotypes (G1 to G6, G8 to G12, and G26) and 16 P genotypes (P[1] to P[8], P[11], P[13], P[19], P[23], P[26], P[27], P[32], and P[34]) of RVA have been associated with pigs (5, 11). However, it is important to note that the distribution and prevalence of specific genotypes can vary across regions and over time. Additionally, genetic variations within the RVA genome, including variations in the NSP4 and VP6 genes, contribute to the overall genetic diversity of the virus. These variations can impact virulence, host range, and the immune response to RVA infection.

Additionally, RVA has the potential for zoonotic transmission and is strong evidence of genetic similarities between human and animal strains, more evident after the full genome sequencing of strains (7, 12, 13). This highlights the importance of ongoing surveillance to assess the extent of transmission and the need for vaccination programs.

Surveillance and molecular characterization of RVA strains in swine populations play a vital role in identifying emerging strains, tracking their spread, and making informed decisions regarding prevention and control measures. However, information on porcine RVA remains limited in Chile, which is also observed for human rotaviruses. Therefore, this study aimed to identify the virus in the swine-intensive population across the country and to characterize porcine RVA through whole genome sequencing (WGS).

## 2. Materials and methods

### 2.1. Sample collection

A total of 154 samples, 86 oral fluids and 68 fecal samples were collected from 1 to 80-day-old pigs between 2015 and 2018. These samples were collected from 22 intensive swine farms located in the central-south area of Chile, including the regions Valparaiso, Metropolitan, O’Higgins, Maule, Ñuble, and Araucanía. This geographical region represents approximately 95% of the national pork production.

Fecal samples were primarily collected to determine intestinal pathogens, hence presented diarrhea, while oral fluids were collected for active surveillance of the Influenza A virus from normal pigs. Fecal samples and oral fluids were collected from different populations. Fecal samples were preserved in 10% phosphate-buffered saline (PBS) containing penicillin, streptomycin, and amphotericin B. Each sample, whether fecal or oral fluid, was subjected to centrifugation at 4,000 rpm for 5 min, followed by aliquoting the supernatant into microtubes containing Minimal Essential Medium at 50% dilution (MEM/EBSS, Cytiva, Hyclone^™^). The MEM was supplemented with 1X TPCK (N-tosyl-L-phenylalanine chloromethyl ketone) Trypsin, 2% Bovine Serum Albumin (BSA-50, Rockland), and 1% antifungal antibiotic solution (Pen-Strep-Amphotericin B solution, 03-033-1B, Biological Industries).

### 2.2. Rotavirus identification and sequencing

To detect RVA, total RNA was extracted from the samples using Chomczynski-phenol solution (Winkler, BM-1,755, Chile) as per the manufacturer’s instructions. Subsequently, a real-time reverse transcription PCR (RT-qPCR) was conducted to amplify a partial region of the VP6 gene, following the protocol described by Marthaler et al. (14). For samples that tested positive, virus isolation was attempted in MA104 cells. Briefly, MA104 cells were cultivated in MEM supplemented with 2% fetal bovine serum (04-127-1A, Biological Industries), and 2% antifungal and antibiotic solution (03-033-1B, Biological Industries) at 37°C with 5% CO_2_. Before infecting the MA104 cell monolayers, the cells were washed with 1X TPCK solution in PBS. Then, 250 μL of the sample was inoculated, and 2 h after inoculation at 37°C with 5% CO_2_, 750 μL of MEM with 2% antifungal, and antibiotic solution was added. The cells were incubated at 37°C with 5% CO_2_ and examined daily for cytopathic effects (CPE) using optical microscopy. At least two passages were performed and isolation was confirmed by RT-qPCR, as previously described (14).

Samples and isolates with RT-qPCR Ct values below 30 were selected for WGS using the Illumina MiSeq platform at the Molecular Diagnostic Development Laboratory, Veterinary Diagnostic Laboratory of the University of Minnesota (MVDL, UMN), USA. Viral RNA was extracted by Qiagen EZ1 nucleic acid extraction using the Virus Mini Kit v 2.0 (Qiagen, Germantown, MD, USA) followed by library preparation using the Takara Bio Clontech SMARTer Stranded total RNA Seq Pico v2 kit (Takara Bio USA, Inc. Mountain View, CA, USA) and then submitted to the University of Minnesota Genomic Centre for sequencing by Illumina Miseq 150 paired-end cycle. The raw fastq files were analyzed using Chan Zuckerberg ID^2^ which is an open-source, free, and cloud-based metagenomics platform (15–17). Briefly, we used the Illumina mNGS Pipeline v8.2, which integrates bioinformatic tools including but not limited to the Spliced Transcripts Alignment to a Reference (STAR) algorithm for initial validation, sequence quality was assessed using FastQC software, and Bowtie2 and HISAT2 to remove remaining host reads. Reads were aligned to the NCBI database using Minimap2 and Diamond, and *de novo* assembling was performed by SPADES. The assembled genomes were for genetic characterization.

To determine the genotypes of the segment sequences, the BV BRC tool for Rotavirus A classification was utilized (18). Phylogenetic analysis was conducted for all segments using reference sequences for each genotype, obtained from the NCBI Virus Variation resource.^3^ Also, other sequences from Chile were incorporated. The sequences were aligned using MUSCLE (19), and the best nucleotide substitution model was determined using JModeltest2 (20). Finally, maximum likelihood trees were estimated for each segment with 1,000 bootstrap replications in MEGA X (21). The construction of phylogenetic trees for each viral segment was performed using the online tool CIPRES^4^ (22) employing a combination of the Maximum Likelihood Tree and Neighbor-Joining Tree. Phylogenetic associations were confirmed using 1,000 bootstrap replicates.

## 3. Results

A total of 154 samples were tested by RT-qPCR, from them 58 (38%) tested positive. In detail, 36 out of 86 oral fluids were positive (41%) and 22 out of 68 fecal samples were positive (32%). Cycle threshold (Ct) values ranged between 14.6 and 33.7, in positive samples fecal samples, the Ct value was significative lower (average 20.3) compared with oral fluids (average 23) (*p* < 0.05). Fourteen out of 22 farms tested positive for RVA at least once, located in all regions in the study. Twenty-one RT-qPCR positive samples (Ct < 30) and two isolates obtained from feces, confirmed by RT-qPCR were selected for NGS, belonging to 9 different farms. The nearly complete genomes of RVA were assembled from virus isolates. Only partial segments with a sequence length between 238 and 606 nt (Supplementary Table S1) were assembled from clinical samples.

All assembled contigs (partial or complete) were analyzed using the Basic Local Alignment Search Tool (BLAST) at NCBI. The closest hits for most of the contigs were found to be related to either swine or human sequences (Table 1; Supplementary Table S1). Among the sequenced isolates, most of the segments showed similarity to porcine RVA strains collected worldwide. Three segments (VP3, NSP2, and NSP5) showed similarity to RVA strains collected from humans. Additionally, all VP2 segments showed similarity (94.2–94.8%) to equine RVA (Table 1). These sequences were submitted to GenBank under the names RVA/Swine/CHL/ROTA-1/2017 and RVA/Swine/CHL/ROTA-2/2017 (Accession numbers OR192577-OR192610).

**Table 1:** Rotavirus A near to complete segments obtained from two isolates and BLAST results.

N	Strain	Sequence length	Name	% Iden	Description	Organism
1	RVA/Swine/Chile/Rota-1/2017VP1	3302	KM820701	94.0%	Rotavirus A isolate RVA/Pig-tc/BEL/RV277/1977/G1P[7] VP1 gene, complete cds	Porcine rotavirus
2	RVA/Swine/Chile/Rota-1/2017VP1	3267	KM820701	94.0%	Rotavirus A isolate RVA/Pig-tc/BEL/RV277/1977/G1P[7] VP1 gene, complete cds	Porcine rotavirus
3	RVA/Swine/Chile/Rota-1/2017VP2	2717	JQ309139	94.1%	Equine rotavirus A strain RVA/Horse-tc/GBR/H-1/1975/G5P9[7] major inner core protein (VP2) gene, complete cds	Equine rotavirus
4	RVA/Swine/Chile/Rota-1/2017VP2	2717	JQ309139	94.2%	Equine rotavirus A strain RVA/Horse-tc/GBR/H-1/1975/G5P9[7] major inner core protein (VP2) gene, complete cds	Equine rotavirus
5	RVA/Swine/Chile/Rota-1/2017VP3	2591	JF796736	92.7%	Porcine rotavirus A strain PRG9121 structural protein VP3 (VP3) gene, complete cds	Porcine rotavirus
6	RVA/Swine/Chile/Rota-1/2017VP3	2508	JF796736	92.5%	Porcine rotavirus A strain PRG9121 structural protein VP3 (VP3) gene, complete cds	Porcine rotavirus
7	RVA/Swine/Chile/Rota-1/2017VP4	2362	JF796737	93.4%	Porcine rotavirus A strain PRG9121 structural protein VP4 (VP4) gene, complete cds	Porcine rotavirus
8	RVA/Swine/Chile/Rota-1/2017NSP1	1572	KJ482251	90.6%	Porcine rotavirus A strain ROTA08 nonstructural protein NSP1 (NSP1) gene, complete cds	Porcine rotavirus
9	RVA/Swine/Chile/Rota-1/2017VP6	1356	AB924099	94.8%	Porcine rotavirus A gene for VP6, complete cds, strain: RVA/Pig-wt/JPN/BU8/2014/G4P[6]	Porcine rotavirus
10	RVA/Swine/Chile/Rota-1/2017VP6	1356	AB924099	94.8%	Porcine rotavirus A gene for VP6, complete cds, strain: RVA/Pig-wt/JPN/BU8/2014/G4P[6]	Porcine rotavirus
11	RVA/Swine/Chile/Rota-1/2017VP7	1035	KT906388	96.9%	Rotavirus A isolate Pig-wt/03/Chi/2013/G5P[7] outer capsid protein (VP7) gene, partial cds	Porcine rotavirus
12	RVA/Swine/Chile/Rota-1/2017VP7	1062	KT906388	96.9%	Rotavirus A isolate Pig-wt/03/Chi/2013/G5P[7] outer capsid protein (VP7) gene, partial cds	Porcine rotavirus
13	RVA/Swine/Chile/Rota-1/2017NSP2	1059	MH267279	95.2%	Porcine rotavirus A isolate RVA/Pig-wt/USA/MN9.65/2008 NSP2 gene, complete cds	Porcine rotavirus
14	RVA/Swine/Chile/Rota-1/2017NSP2	1059	JN104624	96.1%	Human rotavirus A strain Mc345 nonstructural protein (NSP2) gene, complete cds	Human rotavirus
15	RVA/Swine/Chile/Rota-1/2017NSP3	1073	KU739924	91.7%	Porcine rotavirus A strain RVA/Pig-wt/TWN/2–3/2015/G9P13 NSP3 gene, complete cds	Porcine rotavirus
16	RVA/Swine/Chile/Rota-1/2017NSP4	750	JN974789	95.6%	Porcine rotavirus strain F7P4 NSP4 gene, complete cds	Porcine rotavirus
17	RVA/Swine/Chile/Rota-1/2017NSP5	664	AB741659	98.0%	Human rotavirus A NSP5 gene for non-structural protein NSP5, complete cds, strain: Ryukyu-1120	Human rotavirus
18	RVA/Swine/Chile/Rota-1/2017VP1	3302	KM820701	93.6%	Rotavirus A isolate RVA/Pig-tc/BEL/RV277/1977/G1P[7] VP1 gene, complete cds	Porcine rotavirus
19	RVA/Swine/Chile/Rota-2/2017VP1	3267	KM820701	93.9%	Rotavirus A isolate RVA/Pig-tc/BEL/RV277/1977/G1P[7] VP1 gene, complete cds	Porcine rotavirus
20	RVA/Swine/Chile/Rota-2/2017VP2	2717	JQ309139	94.8%	Equine rotavirus A strain RVA/Horse-tc/GBR/H-1/1975/G5P9[7] major inner core protein (VP2) gene, complete cds	Equine rotavirus
21	RVA/Swine/Chile/Rota-2/2017VP3	2591	JF796736	92.9%	Porcine rotavirus A strain PRG9121 structural protein VP3 (VP3) gene, complete cds	Porcine rotavirus
22	RVA/Swine/Chile/Rota-2/2017VP3	2508	KF835911	92.5%	Rotavirus A strain RVA/Human-wt/HUN/BP1792/2004/G4P6/VP3 VP3 gene, complete cds	Human rotavirus
23	RVA/Swine/Chile/Rota-2/2017VP4	2362	JF796737	93.3%	Porcine rotavirus A strain PRG9121 structural protein VP4 (VP4) gene, complete cds	Porcine rotavirus
24	RVA/Swine/Chile/Rota-2/2017NSP1	1551	U08431	91.4%	Porcine rotavirus Gottfried nonstructural protein NSP1 gene, complete cds	Porcine rotavirus
25	RVA/Swine/Chile/Rota-2/2017VP6	1356	U10031	94.4%	Rotavirus CN86 major capsid protein (VP6) RNA, complete cds	Porcine rotavirus
26	RVA/Swine/Chile/Rota-2/2017VP6	1356	AB924099	94.8%	Porcine rotavirus A gene for VP6, complete cds, strain: RVA/Pig-wt/JPN/BU8/2014/G4P[6]	Porcine rotavirus
27	RVA/Swine/Chile/Rota-2/2017NSP3	1073	KU739924	91.7%	Porcine rotavirus A strain RVA/Pig-wt/TWN/2–3/2015/G9P13 NSP3 gene, complete cds	Porcine rotavirus
28	RVA/Swine/Chile/Rota-2/2017VP7	1062	MG066590	92.2%	Porcine rotavirus A strain RVA/Pig-wt/CHN/SCQL-5-2/2017/G3P[13]I5 VP7 (VP7) gene, complete cds	Porcine rotavirus
29	RVA/Swine/Chile/Rota-2/2017VP7	1062	MT874990	92.6%	Porcine rotavirus strain NJ2012 NSP2 protein (NSP2) gene, complete cds	Porcine rotavirus
30	RVA/Swine/Chile/Rota-2/2017NSP2	1059	MH267279	96.1%	Porcine rotavirus A isolate RVA/Pig-wt/USA/MN9.65/2008 NSP2 gene, complete cds	Porcine rotavirus
31	RVA/Swine/Chile/Rota-2/2017NSP2	1059	KT906388	96.9%	Rotavirus A isolate Pig-wt/03/Chi/2013/G5P[7] outer capsid protein (VP7) gene, partial cds	Porcine rotavirus
32	RVA/Swine/Chile/Rota-2/2017NSP4	750	JN974789	95.3%	Porcine rotavirus strain F7P4 NSP4 gene, complete cds	Porcine rotavirus
33	RVA/Swine/Chile/Rota-2/2017NSP5	664	AB741659	98.0%	Human rotavirus A NSP5 gene for non-structural protein NSP5, complete cds, strain: Ryukyu-1120	Human rotavirus

The pairwise comparison of RVA/Swine/CHL/ROTA-1/2017 and RVA/Swine/CHL/ROTA-2/2017 segments reported an identity of 80.3 to 100% between them. Duplicate segments were collected from both isolates for some segments. In detail, for VP1 four segments were obtained, with an identity of 97.8 to 99.9%; for VP2, three segments with an identity of 97.6 to 99.9%; for VP3, four segments with an identity of 89.5 to 99.6%; for VP4 has two segments with an identity of 99.8% between them; for NSP1, two segments with an identity 87.4%; for VP6, four segments with an identity of 92.2 to 99.6%; for VP7, four segments with an identity of 80.3 to 100%; for NSP2, four segments with an identity of 87.2 to 99.7%; for NSP3, two identical segments; for NSP4, three segments with an identity of 99.4 to 100%; and NSP5 has two identical segments. The BV-BRC tool classified all internal segments as I5-R1-C1-M1-A8-N1-T1-E1-H1, which are genetically related to viruses collected from humans and swine. Using VP7 and VP4 genotyping the isolate ROTA-1 was classified as G5-P[7] and the isolate ROTA-2 presented G5-P[7] and G3-P[7] evidencing double infection.

**Figure 1:**
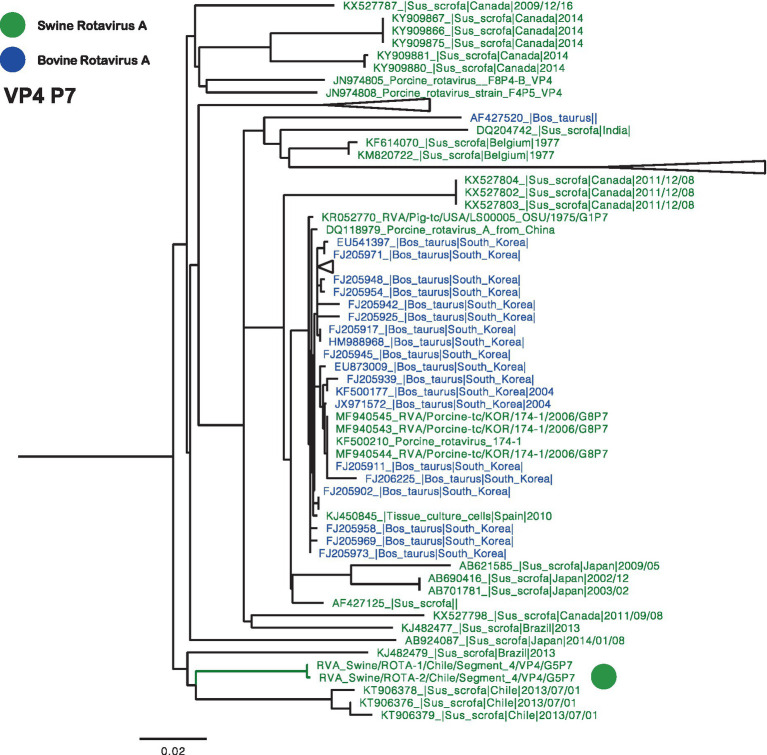
Phylogenetic tree of Rotavirus A segment 4 (VP4), P[7] genotype. The final dataset included 104 sequences. The Chilean sequences are depicted with a green dot. Strains are highlighted in colors: Swine RVA (Green), and Bovine (Blue).

Phylogenetic trees were constructed for all segments, and partial sequences were excluded from the analysis. The VP4 phylogeny, specifically genotype P[7], revealed that Chilean RVA strains formed a monophyletic cluster, which also included previously reported strains from 2013 (Figure 1) (23). In addition to closely related Chilean sequences, similar viruses have been identified in swine and bovine populations across different regions globally.

**Figure 2:**
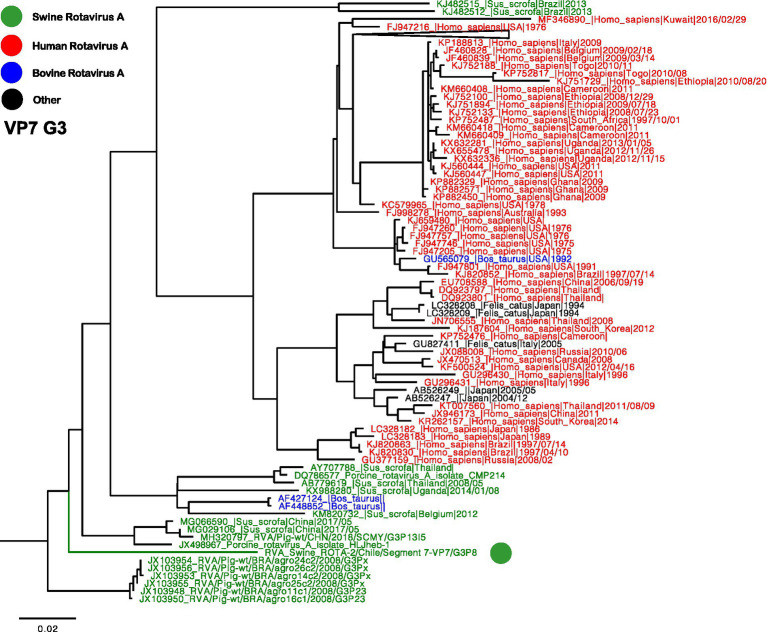
Phylogenetic tree of Rotavirus A segment 9 (VP7), G3 genotype. The final dataset included 371 sequences. The Chilean sequence is depicted with a green dot. Strains are highlighted in colors: Human RVA (Red), Swine RVA (Green), Bovine (Blue), and other origins in black.

**Figure 3:**
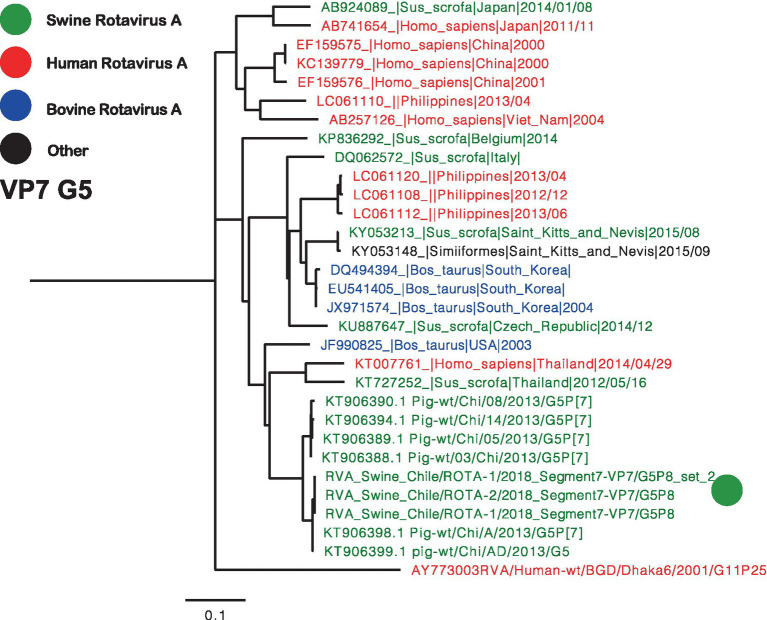
Phylogenetic tree of Rotavirus A segment 9 (VP7), G5 genotype. The final dataset included 31 sequences. The Chilean sequences are depicted with a green dot. Strains are highlighted in colors: Human RVA (Red), Swine RVA (Green), Bovine (Blue), and another origin in black.

Two separate trees were generated for VP7, representing genotypes G3 and G5 (Figures 2, 3). Notably, the G3 genotype has never been documented in Chile before, making the Chilean strain a unique singleton that shares its origin with strains found in humans, swine, and bovines (Figure 2). On the other hand, the remaining three segments are classified as G5, and the phylogenetic analysis grouped the Chilean sequences into a monophyletic cluster, including the partial sequences reported in 2013 (Figure 3) (23). These viruses have a common origin with strains detected in swine, humans, and bovines from various parts of the world. In the case of related sequences detected in humans, those detected in the Philippines have been associated with zoonotic detections (24).

The phylogenies for the rest of the genes can be divided into two groups. The phylogenies for VP1, VP2, NSP2, NSP3, NSP4, and NSP5 grouped the Chilean sequences in monophyletic clusters, while the phylogenies for VP6, VP3, NSP1 evidence two different origins of the Chilean sequences (Supplementary Figures S1–S9). In both cases, most of the sequences are more closely related to swine and human RVA sequences reported worldwide. For VP1 phylogeny the closest sequences that have been reported from swine in Belgium have been related to zoonotic events (25). For VP2 the closest sequence belonged to an ancient equine strain RVA/Horse-tc/GBR/H-1/1975/G5P9[7] (JQ309139). The NSP2 phylogeny grouped the Chilean sequences in a monophyletic group and related with human and swine sequences. The NSP3 phylogeny grouped the Chilean sequences with RVA detected in humans from Argentina, Ecuador, and Brazil. For NSP4 the Chilean sequences are more closely with RVA detected in 2006–2007 in swine from Canada (26). The NSP5 sequences are related to swine RVA reported from South Africa. On the other hand, the VP3 phylogeny clustered Chilean sequences into two groups and both groups related to human origin RVA sequences, like NSP1 and VP6 but in this case more closely related to RVA from swine (Supplementary Figures S1–S9).

## 4. Discussion

Group A rotaviruses are considered important viral agents that cause gastroenteric problems in intensive swine production centers, mainly affecting young pigs (27), and commonly detected in intensive production farms worldwide (28–30). In the present study, RVA was identified in 14 out of 22 intensive farms, confirming the widespread of RVA in Chilean farms, which was first reported in the country in 1983 (31). In South America, RVA has been reported in pigs in most countries such as Argentina, Peru, Brazil, and Colombia (32–35).

Rotavirus A has been identified in both feces and oral fluids, with its presence in feces being attributed to the well-established pathogenicity of the virus (5). The detection of the virus in oral fluids can be primarily attributed to the composition of these fluids in pigs, which may contain remnants of feces (36). However, recently (37) conducted experiments using a murine model, revealing that rotavirus can replicate within salivary glands and spread via saliva (37). This evidence implies the potential for viral excretion through saliva in swine, which should be further investigated.

Rotavirus A exhibits significant genetic diversity, with various genotypes capable of affecting pigs, other animals, and even humans. To date, there are 58 genotypes for P, 42 for G, 39 genotypes for A, 32 genotypes for I and E, 28 genotypes for R, N, T, and H, and 24 genotypes for C and M (RCWG, 2023). This extensive variation necessitates a comprehensive genome characterization to gain a better understanding of the epidemiology and zoonotic potential of the virus. In this study, we conducted a thorough investigation into the viral genome of porcine-origin RVA by employing WGSof isolates and clinical samples. While the full genome sequencing of isolates was successful, we encountered challenges in WGS directly from clinical samples, including fecal and oral fluids. These discrepancies may be attributed to the presence of other agents in direct samples that might have dominated the sequencing run, thus hindering the detection of RVA. We initially viewed the utilization of direct samples for NGS as a promising alternative, given the low Ct values observed in the samples used. However, our findings indicate that this approach is not recommended using the current protocol. One possible explanation for this limitation is the challenge of effectively denaturing double-stranded RNA (dsRNA). Recent reports have highlighted the use of dimethyl sulfoxide (DMSO) as a potential solution to improve dsRNA virus sequencing (38). Another alternative approach could involve preamplification through PCR followed by NGS, as demonstrated by Nyaga et al. (39), but this process is time-consuming. These results are interesting for future studies to improve the NGS attempting RVA sequencing from direct samples.

We were able to obtain the nearly complete genome of RVA from two isolates. The two outer capsid proteins VP7 (G) and VP4 (P) sequenced were more closely related to isolates previously detected in swine. We obtained G5 and G3 genotypes for VP7 and P[7] for VP4, common combinations reported previously (40). The G5 and G3 genotypes are usually detected in pigs, humans, and cattle (41, 42). The G5P[7] genotype combination is the most commonly detected in swine in the Americas (5), thus our findings are consistent with previous reports. Notably, we detected the presence of duplicated segments, including G5 and G3, in the isolates, indicating the potential occurrence of co-infection or mixed infection. This observation is particularly interesting as these samples were collected from individual animals, supporting the possibility of multiple viral strains circulating within the same host (28).

For the remaining segments, we identified the genotypes I5-R1-C1-M1-A8-N1-T1-E1-H1. While genotypes R1-C1-M1-N1-T1-T1-E1-H1 are directly related to human-origin RVA, I5, and A8 are more related to swine-origin RVA (11). Then, we reported constellation G5/G3-P [7] -I5-R1-C1-M1-A8-N1-T1-E1-H1 which is similar to other constellations obtained from swine samples from different countries (25). Using the phylogenetic trees, we established the genetic relationship between Chilean RVA strains and viruses found in swine, humans, and bovine populations across different parts of the world. These results can suggest the potential role of RVA as a zoonotic and/or reverse zoonotic pathogen, capable of reassortment due to its segmented nature (43). It is important to note that the reassortment events of Rotavirus A occur frequently with RVA strains from the same animal species but can also occur with strains originating from animals and humans (44–46). Examples of genetic reassortment of RVA strains from swine to human are the introductions of the swine G9 (11) and the P[6] to human strains (47, 48). Additionally, a case of G5 genotype reassortment between swine and human viruses was reported in children from Brazil, Paraguay, and Argentina (7).

In general, BLAST reported <95% identity of Chilean sequences compared to reference sequences indicating a substantial level of genetic divergence. This high divergence can be attributed to the limited availability of RVA sequences for comparison purposes and phylogeny. The epidemiological significance of our findings may be influenced by the lack of sufficient data. It is important to note that even in Chile, there is a limited information of publicly available human RVA sequences, with only partial VP7 sequences accessible for analysis.

The results obtained from this study suggest that the viruses identified and sequenced may have originated from reassortments of RVA originating from humans, swine, and other animal species. Therefore, improving the NGS for dsRNA viruses such as RVA is mandatory in Chile and other countries. In summary, while the present study provides valuable insights into the genetic diversity and potential origins of RVA, further research is necessary to fully comprehend its complex epidemiology.

## Data availability statement

The datasets presented in this study can be found in online repositories. The names of the repository/repositories and accession number(s) can be found at: https://www.ncbi.nlm.nih.gov/ OR192577-OR192610.

## Ethics statement

The animal study was approved by all procedures involving animals were approved by the Institutional Committee for Animal Care and Use (CICUA) of the University of Chile under certificate number 02-2016. The study was conducted in accordance with the local legislation and institutional requirements.

## Author contributions

VN, SM, and BB-R contributed to the conception and design of the study. CM, VV, BB-R, and VN performed the analysis. CM, CU-E, GR-T, and VN wrote the first draft of the manuscript. All authors contributed to the manuscript revision and approved the submitted version.

## Footnotes

^1^https://rega.kuleuven.be/cev/-viralmetagenomics/virus-classification/rcwg, Accessed May 30, 2023.

^2^https://czid.org/

^3^https://www.ncbi.nlm.nih.gov/genomes/VirusVariation/-Database/nph-select.cgi?taxid=28875

^4^www.phylo.org
