# Supplementary material for: Identification and characterization of porcine Rotavirus A in Chilean swine population
Source: Front Vet Sci. 2023 Nov 2;10:1240346. doi: 10.3389/fvets.2023.1240346 (PMC10652281; doi:10.3389/fvets.2023.1240346)
Supplement: Supplementary file 1 [file Data_Sheet_1.docx]

Supplementary Figure 1. Phylogenetic tree of Rotavirus A segment 1 (VP1 pol), R1 genotype. The final dataset included 254 sequences. The Chilean sequences are depicted with a green dot. Strains are highlighted in colors: Human RVA (Red), Swine RVA (Green), Bovine (Blue), and another origin in black.

Supplementary Figure 2. Phylogenetic tree of Rotavirus A segment 2 (VP2), C1 genotype. The final dataset included 143 sequences. The Chilean sequences are depicted with a green dot. Strains are highlighted in colors: Human RVA (Red), Swine RVA (Green), Bovine (Blue), Equine, (Purple), and another origin in black.

Supplementary Figure 3. Phylogenetic tree of Rotavirus A segment 3 (VP3), M1 genotype. The final dataset included 257 sequences. The Chilean sequences are depicted with green dots. Strains are highlighted in colors: Human RVA (Red), Swine RVA (Green), Bovine (Blue), and Equine (Purple).

Supplementary Figure 4. Phylogenetic tree of Rotavirus A segment 5 (NSP1), A8 genotype. The final dataset included 36 sequences. The Chilean sequences are depicted with green dots. Strains are highlighted in colors: Human RVA (Red), Swine RVA (Green), and another origin in black.

Supplementary Figure 5. Phylogenetic tree of Rotavirus A segment 6 (VP6), I5 genotype. The final dataset included 36 sequences. The Chilean sequences are depicted with green dots. Strains are highlighted in colors: Human RVA (Red), Swine RVA (Green), and another origin in black.

Supplementary Figure 6. Phylogenetic tree of Rotavirus A segment 8 (NSP2) genotype N1. The final dataset included 1,413 sequences. The Chilean sequences are depicted with a green dot. Strains are highlighted in colors: Human RVA (Red), Swine RVA (Green), and another origin in black.

Supplementary Figure 7. Phylogenetic tree of Rotavirus A segment 7 (NSP3) genotype T1. The final dataset included 1,293 sequences. The Chilean sequences are depicted with a green dot. Strains are highlighted in colors: Human RVA (Red), and Swine RVA (Green).

Supplementary Figure 8. Phylogenetic tree of Rotavirus A segment 10 (NSP4) E1 genotype. The final dataset included 868 sequences. The Chilean sequences are depicted with a green dot. Strains are highlighted in colors: Human RVA (Red), and Swine RVA (Green).

Supplementary Figure 9. Phylogenetic tree of Rotavirus A segment 11 (NSP5) H1 genotype. The final dataset included 1,255 sequences. The Chilean sequences are depicted with a green dot. Strains are highlighted in colors: Human RVA (Red), Swine RVA (Green), and Equine RVA in purple.

Supplementary information.

Table 1. Rotavirus A contigs, which were obtained by NGS from five direct samples and BLAST results.
